# Acute kidney injury in an intensive care unit of a general hospital with emergency room specializing in trauma: an observational prospective study

**DOI:** 10.1186/s12882-015-0026-4

**Published:** 2015-03-19

**Authors:** Paulo Roberto Santos, Diego Levi Silveira Monteiro

**Affiliations:** Graduate Program in Health Sciences, Sobral Faculty of Medicine, Federal University of Ceará, Brazil, Rua Comandante Maurocélio Rocha Ponte 100, Sobral, CEP 62.042-280 Brazil

**Keywords:** Acute kidney injury, Intensive care unit, Trauma

## Abstract

**Background:**

Acute kidney injury (AKI) is common among intensive care unit (ICU) patients and is associated with high mortality. Type of ICU, category of admission diagnosis, and socioeconomic characteristics of the region can impact AKI outcomes. We aimed to determine incidence, associated factors and mortality of AKI among trauma and non-trauma patients in a general ICU from a low-income area.

**Methods:**

We studied 279 consecutive patients in an ICU during a follow-up of one year**.** Patients with less than 24-hour stay in the ICU and with chronic kidney disease were excluded. AKI was classified according to the Kidney Disease Improving Global Outcomes (KDIGO) criteria in three stages. Comparisons were performed by the Student-t and Mann–Whitney tests for continuous variables, respectively with and without normal distribution. Comparisons of frequencies were carried out by the Fisher test. Multivariate logistic regression was used to test variables as predictors for AKI and death.

**Results:**

Admission categories were proportionally divided into 51.6% of non-trauma diagnosis and 48.4% of trauma cases. Most trauma cases involved brain injury (79.5%). The overall incidence of AKI was 32.9%, distributed among the three stages: 33.7% stage 1, 29.4% stage 2 and 36.9% stage-3. Patients who developed AKI were older, had more diabetes, stayed longer in the ICU, presented higher APACHE II and more often needed mechanical ventilation and use of vasopressors. In comparison with non-trauma cases, trauma patients had a greater prevalence of males, higher APACHE II score, higher urine output, and younger age. There was no difference concerning development of AKI and crude mortality between trauma and non-trauma patients. Age, presence of diabetes, APACHE score and use of vasopressors were independent predictors for AKI, and AKI increased the risk of death ten-fold (OR = 14.51; CI 95% = 7.94-26.61; p < 0.001).

**Conclusions:**

There was a high incidence of AKI in this study. AKI was strongly associated with mortality both among trauma and non-trauma patients. Trauma cases, especially brain injury due to traffic accidents involving motorized two-wheeled vehicles, should be seen as an important preventable cause of AKI.

## Background

Acute kidney injury (AKI) is a global health problem [1]. Incidence and outcomes of AKI are well known for hospitalized patients [2]. However, incidence, risk and protective factors for AKI vary according to socioeconomic status in different regions of the world and also according to hospital facilities. The literature contains fewer studies on AKI among hospitalized patients from developing than from developed countries. And particularly there is a lack of data about community-acquired AKI in developing countries. In these countries, there is a double challenge, comprising not only the diagnosis and treatment of the more typical hospital cases of AKI due to clinical and surgical complications, but also specific aspects of community-acquired AKI in poor regions, like diarrheal diseases, leptospirosis, dengue, animal venoms, obstetric complications etc. [3-5].

Trauma accounts for 15% of ICU admissions in the United States [6]. In developing countries, trauma is a well-recognized emerging cause of ICU admission (community-acquired AKI in developing countries), affecting mainly young and previously healthy people. Like in other low- and middle-income areas of the globe, in Sobral, a city located in the north region of the state of Ceará, northeast Brazil, we are witnessing an exponential increase of traffic accidents, especially involving motorcycles/scooters. This is due to the recent improvement of people’s standard of living, bringing large-scale replacement of bicycles with motorized two-wheel vehicles. An aggravating factor for severity of this kind of accident is the widespread failure to use helmets, leading to an epidemic of brain injury [7].

In Sobral, medical response to trauma cases is concentrated in a single hospital (Santa Casa de Misericórdia de Sobral Hospital), where the most serious cases, attended first in the emergency room, are transferred to the ICU. This ICU is of the general type since it receives trauma patients coming from the emergency room and also patients coming from clinical and surgical wards of the hospital. Therefore, the profile of this ICU offers a great opportunity to study AKI in only one set, where typical non-trauma cases of AKI coexist with AKI developed among trauma patients, and to test a previous and not yet confirmed hypothesis of trauma being a protective factor for AKI due to the fact it affects younger and healthier people.

Thus, stimulated by (i) the lack of data on AKI epidemiology and outcomes in developing countries, (ii) the fact that trauma has become an important cause of community acquired AKI in several developing areas of the world, and (iii) the availability of an ICU treating trauma and non-trauma cases, we aimed to determine the incidence, associated factors and mortality among trauma and non-trauma patients with AKI in an ICU from a developing area.

## Methods

The study setting was an ICU of a tertiary hospital, named Santa Casa de Misericórdia de Sobral Hospital, which is a referral facility for several towns within an area of 34,560 km^2^ (37.3 inhabitants/ km^2^) in the northern region of Ceará state, northeast Brazil. The unit is also a training venue for interns. This hospital has medical specialties in the clinical field (mainly nephrology, pneumology, rheumatology and hematology) and surgical area (general and neurosurgery), along with an emergency room specializing in trauma. All trauma cases from the region described above needing ICU assistance are referred to this hospital. Written informed consent was obtained from participants or their appropriate surrogates, and the study protocol was approved by the ethics committee of Vale do Acaraú University, with which the hospital is associated.

We studied 279 consecutive patients admitted to the ICU during a follow-up of one year (May 2013 to April 2014). Patients with less than 24-hour stay in the ICU and with chronic kidney disease were excluded. Chronic kidney disease was excluded based on medical records and/or laboratory results showing glomerular filtration rate less than 60 mL/min per 1.73 m^2^ at least 3 months before ICU admission. We collected demographic and clinical data from all patients, such as gender, age, category of ICU admission diagnosis, length of ICU stay (in days), classification by Acute Physiology and Chronic Health Evaluation II (APACHE II) and need for diuretics, mechanical ventilation, vasoactive therapy and renal replacement therapy (RRT). Admission diagnoses were classified into main two groups: trauma and non-trauma. Trauma cases were divided according to the main mechanism of trauma into brain injury, chest trauma, abdominal trauma and musculoskeletal trauma. Non-trauma cases were divided into five categories: neurologic, respiratory, cardiovascular, sepsis and miscellaneous. Examples of these include cerebral tumors and aneurysms, ketoacidosis, exogenous intoxication, surgical and obstetric complications, pancreatitis and pneumonia. Furosemide was the only diuretic used. Vasoactive therapy comprised dopamine, noradrenaline and/or dobutamine. RRT was conventional hemodialysis for all patients who were submitted to dialysis. Continuous methods of hemodialysis or peritoneal dialysis were not available. As an observational study, our research did not affect any medical prescription. Creatinine and diuresis volume were evaluated daily. AKI was classified according to the Kidney Disease Improving Global Outcomes (KDIGO) criteria into three stages [8]. The lowest creatinine level known through medical records was used to determine the change in creatinine. In the case of unknown previous creatinine value, we considered the lowest creatinine value during the stay in the ICU as baseline. The highest creatinine level during ICU stay was used in comparisons between groups. Patient weight for determination of diuresis volume (mL/kg/h) was estimated because there was no facility for weighing the patients in the ICU. Outcomes were death during ICU stay and discharge from the ICU. There were five transfers of patients to other ICUs, which were considered as discharge.

Data are mean ± SD for continuous variables with normal distribution, median, minimum and maximum values for continuous variables without normal distribution and absolute value and percentage for frequency of categorical variables. Comparisons were performed by the Student-t and Mann–Whitney tests for continuous variables, respectively with and without normal distribution. Comparisons of frequencies were carried out by the Fisher test. Simple (unadjusted) and multivariate (adjusted) logistic regression was performed to test variables as predictors of AKI and death. Variables tested in logistic regression were those that differed in the comparison between patients with and without AKI and between survivors and non-survivors. Trauma was tested as predictor of AKI and death although it did not differ in the comparisons.

## Results

Sample characteristics are shown in Table 1. Half the patients were trauma cases and half were non-trauma cases. The overall incidence of AKI was 32.9%, proportionally distributed through the three stages: 33.7% stage 1 (8 according to urine output; 21 according to creatinine change; 2 according to both criteria), 29.4% stage 2 (3 according to urine output; 22 according to creatinine change; 2 according to both criteria) and 36.9% stage 3 (1 according to urine output; 12 according to creatinine change; 21 according to both criteria). Although there were 92 cases of AKI, only 20 patients were submitted to RRT: 1 of 31 in stage 1, 1 of 27 in stage 2, and 18 of 34 in stage 3. Overall mortality was 33.3%.

**Table 1:** **Sample characteristics**

**Variables**	
**Gender**	
Male	185 (66.3)
Female	94 (33.7)
**Age** (years)	42,5 ± 20,7
**Category of admission diagnosis**	
Trauma	135 (48.4)
Neurologic	63 (22.6)
Respiratory	24 (8.6)
Cardiovascular	9 (3.2)
Sepsis	8 (2.9)
Miscellaneous	40 (14.3)
**Diabetes**	36 (12.9)
**Length of stay** (days)	8,1 ± 7,0
**APACHE II**	8 (0–28)
**Diuretic use**	109 (39.0)
**Mechanical ventilation**	182 (65.2)
**Vasoactive therapy**	74 (26.5)
**Renal replacement therapy**	20 (7.2)
**Creatinine** (mg/dL)	1.4 ± 1.4
**Diuresis** (mL)	3,091 ± 1,666
**Acute Kidney Injury**	
Yes	92 (32.9)
No	187 (67.1)
**Acute Kidney Injury Stages**	
1	31 (33.7)
2	27 (29.4)
3	34 (36.9)
**Death**	
Yes	93 (33.3)
No	186 (66.7)

Data are means ± SD and percentages in parentheses, except for APACHE II: data are median and minimum and maximum values in parentheses.

Patients who developed AKI were older, had more diabetes, stayed longer in the ICU, had higher APACHE II score, and were more often submitted to mechanical ventilation and more frequently under use of vasopressors (Table 2). In the multivariate analysis, development of AKI was predicted by age, diabetes, APACHE II score and use of vasoactive drugs (Table 3). Mortality among patients with AKI was 71.7% in contrast to 14.4% mortality among patients without AKI (Table 2).

**Table 2:** **Comparison between patients with and without acute kidney injury**

**Variable**	**Without acute kidney injury**	**With acute kidney injury**	**P**
	**N = 187**	**N = 92**	
**Gender**			
Male	122 (65.3)	63 (68.5)	0.894
Female	65 (34.7)	29 (31.5)	
**Age** (years)	39.9 ± 16.6	47.6 ± 22.0	0.003
**Category of admission diagnosis**			
Trauma	94 (50.2)	41 (44.5)	0.376
Non-trauma	93 (49.8)	51 (55.5)	
**Diabetes**	17 (9.0)	19 (20.6)	0.025
**Length of stay** (days)	7.4 ± 5.5	9.5 ± 9.2	0.021
**APACHE II**	7 (0–21)	10 (0–28)	<0.001
**Diuretic use**	70 (37.4)	31 (33.6)	0.596
**Mechanical ventilation**	112 (59.9)	69 (75.0)	0.016
**Vasoactive therapy**	36 (19.2)	37 (40.2)	<0.001
**Renal replacement therapy**	0	20 (21.7)	<0.001
**Creatinine** (mg/dL)	0.7 ± 0.1	2.6 ± 2.0	<0.001
**Diuresis** (mL)	3,657 ± 1,331	2,004 ± 1,711	<0.001
**Death**			
Yes	27 (14.4)	66 (71.7)	<0.001

Data are means ± SD and percentages in parentheses, except for APACHE II: data are median and minimum and maximum values in parentheses.

**Table 3:** **Logistic regression for risk of acute kidney injury**

**Variable**	**Unadjusted**		**Adjusted**	
	**OR (95% CI)**	**P**	**OR (95% CI)**	**P**
**Age**	1.01 (1.01-1.03)	0.004	1.01 (1.00-1.03)	0.011
**Diabetes**	2.53 (1.25-5.16)	0.010	2.19 (1.05-4.59)	0.037
**APACHE II**	1.11 (1.06-1.17)	<0.001	1.11 (1.06-1.17)	<0.001
**Mechanical ventilation**	1.11 (0.82-1.53)	0.485	0.97 (0.69-1.38)	0.899
**Vasoactive therapy**	2.64 (1.53-4.58)	<0.001	2.52 (1.45-4.54)	0.002
**Trauma**	0.74 (0.45-1.23)	0.253	0.91 (0.56-1.65)	0.756

Most cases of trauma were classified as traumatic brain injury (79.5%), followed by chest trauma (7.9%), abdominal trauma (7.2%) and musculoskeletal trauma (5.4%). In the comparison of patients with and without trauma, trauma patients had a higher prevalence of males, were younger, had higher APACHE II score and higher urine output (Table 4). There was no difference concerning development of AKI and crude mortality between trauma and non-trauma patients (Table 4); likewise trauma did not affect the development of AKI or death (Tables 3 and 5).

**Table 4:** **Comparison between trauma and non-trauma patients**

**Variables**	**Trauma N = 137**	**Non-trauma N = 142**	**P**
**Gender**			
Male	110 (80.9)	74 (52,2)	<0.0001
Female	27 (19.1)	68 (47.8)	
**Age** (years)	34.9 ± 16.4	50.0 ± 21.0	<0.0001
**APACHE II**	10 (0–28)	7 (0–25)	0.040
**Creatinine** (mg/dL)	0.8 ± 1.7	0.9 ± 1.4	0.457
**Diuresis** (mL)	3,313 ± 1,529	2,870 ± 1,770	0.028
**Acute kidney injury**	41 (28.6)	52 (36.6)	0.309
**Death**	39 (28.6)	55 (31.7)	0.099

Data are means ± SD and percentages in parentheses, except for APACHE II: data are median and minimum and maximum values in parentheses.

**Table 5:** **Logistic regression for risk of death**

**Variable**	**Unadjusted**		**Adjusted**	
	**OR (95% CI)**	**P**	**OR (95% CIcp)**	**P**
**AKI**	14.51 (7.94-26.6)	<0.001	10.34 (5.47-19.57)	<0.001
**Vasoactive therapy**	3.67 (2.10-6.45)	<0.001	2.91 (1.47-5.77)	0.002
**Mechanical ventilation**	4.59 (2.43-8.69)	<0.001	1.13 (0.72-1.79)	0.596
**APACHE II**	1.09 (1.05-1.15)	<0.001	1.07 (1.01-1.14)	0.016
**Age**	1.01 (1.00-1.03)	0.010	0.97 (1.00-1.03)	0.113
**Trauma**	0.53 (0.32-0.92)	0.022	0.64 (0.33-1.29)	0.215

AKI was an independent risk of death, showing an adjusted hazard ratio of 10.34, along with vasoactive therapy (OR = 2.91) and APACHE II score (OR = 1.07) (Table 5).

## Discussion

We believe the ICU in our study is an ideal setting to investigate AKI in a mixed sample comprising trauma (an emerging cause of AKI in developing countries) and non-trauma cases, creating an opportunity to describe the scope of AKI in such a general ICU, and furthermore detect differences in incidence and outcomes between AKI associated with clinical and surgical complications and AKI affecting healthy subjects who were victims of trauma. Our results showed an overall high incidence of AKI but did not point to any difference regarding incidence and outcomes of AKI between trauma and non-trauma patients, and confirmed the great impact of AKI on ICU patients’ mortality.

Before the widespread use of consensus definitions of AKI, incidence of AKI in ICUs was reported to be low [9], probably due to the fact that previous definitions of AKI usually did not consider urine output. After incorporation of currently available methods of classification, such as KDIGO, RIFLE and AKIN (all three based on creatinine changes and/or decrease of urine output), the overall incidence of 32.9% of AKI in our study is within the range of 16% to 39% found in the literature [10,11]. When looking separately at the incidence of AKI among trauma and non-trauma cases, the incidence of 36.6% among non-trauma patients is within the range shown above, although the AKI incidence of 28.6% among trauma patients is above the range of 6% to 17.3% found in the literature [6,12,13]. An explanation could be that trauma patients were more often severely ill, according to their higher APACHE II score compared to non-trauma cases. Another possible interpretation is that most cases of trauma in our sample were brain injury, which is a kind of trauma that directly affects the kidneys by reducing glomerular perfusion. Additionally, brain injury is associated with more frequent use of mechanical ventilation and longer ICU stay [14-16].

Our hypothesis was that trauma affecting younger and healthier subjects could be less associated with AKI. Indeed, the idea that trauma compared to other causes of ICU admission is less associated with AKI has appeared in previous studies [15]. In our study, all hypotheses of trauma being a protective factor for AKI among ICU patients were rejected. But we have to point out that our results can be due to the fact that non-trauma patients of our sample were not severely ill, based on relatively low mean APACHE II score, as well as the few cases of sepsis and many elective admissions after neurosurgery. Trauma was only a protective factor for death in the univariate analysis (but not in the multivariate model). The differences between trauma and non-trauma cases were those widely expected: trauma patients comprised younger patients with more men than women.

Trauma is included in the list of modifiable risk factors for AKI, alongside sepsis, exposure to radiocontrast media and drug toxicity. We think the AKI incidence of 28.6% among trauma patients is alarming. It indicates that a large portion of trauma patients are dying due to development of AKI. In our sample, most trauma cases were brain injury, which in our region is strongly associated with traffic accidents involving motorcycle/scooter riders not using helmets, like in other developing areas [17]. Thus, prevention of AKI in such areas could be achieved based on regional campaigns addressing cultural habits like considering motorcycles/scooters to be toys (as demonstrated by the wide use among young teens), overcapacity (more than two riders), backseat passengers without helmets, lack of regulation of motorcycle taxis and alcohol abuse by riders. In addition, there are specific approaches to prevent AKI when brain injury occurs, like intracranial pressure monitoring to reduce the use of mannitol (known for posing a risk of AKI in brain injury patients) [18]. Intracranial pressure monitoring is not routinely implemented in the ICU studied.

There was no surprise about factors associated with AKI found: older age, diabetes, higher APACHE II score, lower urine output, and use of vasopressors. Three findings about associated factors deserve explanation. First, the smaller number of cases of AKI among patients with neurologic disease (non-trauma) is certainly due to the presence of a neurology department in the hospital whose patients are routinely admitted to the ICU after neurosurgery. Since most of these patients were submitted to elective surgery, they were more clinically stable. Second, the same fact explains the high incidence of mechanical ventilation, which is routinely implemented during the post-neurosurgery period. In addition, it also explains the lack of association of mechanical ventilation with AKI and death (mechanical ventilation only predicted death in the univariate analysis), since elective neurosurgery patients submitted to mechanical ventilation were healthier. Third, the low use of vasopressors in the sample is clearly due to the few cases of sepsis.

The difference of 14.4% in mortality in patients without AKI against 71.7% among patients with AKI is emblematic. As expected, AKI was a powerful and independent predictor, increasing the risk of death ten-fold. At the beginning of the study, one of our aims was to compare mortality according submission or not to RRT between the three stages of AKI. This would be worthwhile since there is no consensus on when to start RRT in the early stages of AKI, so studies testing the effects of RRT on mortality in early stages are welcome. However, this aim was not met due to the very few number of patients submitted to RRT in stages 1 and 2 (one patient from each stage). In fact, it was surprising that only 18 patients among 34 with AKI of stage 3 were submitted to RRT. As an observational study, we did not interfere with any kind of medical prescription or management. The systematic method to classify AKI (as used in our study) is not routinely performed in the ICU. We can suppose that the lack of systematic criteria for screening AKI in the ICU could have lead to under-diagnosis of AKI by the ICU team. We cannot discard the possibility that under-diagnosis and less indication of RRT influenced the high mortality associated with AKI in the sample.

### Limitations

We are aware of the study’s limitations. First, our findings cannot be extrapolated to more typical ICU samples with more cases of sepsis. Indeed, our setting is very specific, with half trauma and half non-trauma cases, consequently with few cases of sepsis, which is the main cause of AKI in more typical ICUs. Thus, our results may not apply to other general ICUs. Nonetheless, we think this characteristic of our setting was useful in studying possible protective factors of trauma in provoking AKI. Second, as an observational study we collected the routine data available in the ICU. Only two kinds of data were not routinely available and were generated by us: KDIGO and admission diagnosis categories. Thus, some variables are missing that could enrich the study, for example the Injury Severity Score and creatine phosphokinase test. Even with many trauma cases, the setting was a general ICU where score for trauma is not routinely applied. The Injury Severity Scale could be more appropriate than APACHE among trauma patients. Also among trauma patients, creatine phosphokinase testing would be interesting to indicate rhabdomyolysis. Third, there were no facilities to weigh patients in the ICU and urine output by weight was estimated (as routinely done in the ICU of our study to calculate drug doses). While we recognize this as a limitation, we do not believe this method misclassified patients according to AKI stages. Finally, we are aware that the small size of the sample brings risk of type II statistical error.

## Conclusion

There was a high incidence of AKI in this study. AKI was strongly associated with mortality both among trauma and non-trauma patients. Trauma cases, especially brain injury due to traffic accidents involving motorcycles/scooters should be seen as an important preventable cause of AKI.

## Abbreviations

AKI: Acute kidney injury
AKIN: Acute Kidney Injury Network
APACHE II: Acute Physiology and Chronic Health Evaluation II
ICU: Intensive care unit
KDIGO: Kidney Disease Improving Global Outcomes
RIFLE: Risk Injury Failure Loss of kidney function and End-stage kidney disease
RRT: Renal replacement therapy

## References

[1] Li PKT, Burdmann EA, Mehta R (2013). Acute kidney injury: global alert. Kidney Int.

[2] Uchino S, Kellum JA, Bellomo R, Doig GS, Morimatsu H, Morgera S (2005). Acute renal failure in critically ill patients. JAMA.

[3] Lombardi R, Yu L, Younes-Ibrahim M, Schor N, Burdmann EA (2008). Epidemiology of acute kidney injury in Latin America. Semin Nephrol.

[4] Naicker S, Aboud O, Ghrbi MB (2008). Epidemiology of acute kidney injury in Africa. Semin Nephrol.

[5] Jayakumar M, Prabahar MR, Fernando EM, Manorajan R, Venkatraman R, Balarman V (2006). Epidemiologic trend changes in acute renal failure: a tertiary center experience from South India. Ren Fail.

[6] Podoll AS, Kozar R, Holcomb JB, Finkel KW (2013). Incidence and outcome of erly acute kidney injury in critically-ill trauma patients. PLoS One.

[7] Albuquerque CEL, Arcanjo FPN, Cristino-Filho G, Lopes-Filho AM, de Almeida PC, Prado R (2014). How safe is your motorcycle helmet?. J Oral Maxillofac Surg.

[8] Kidney Disease Improving Global Outcomes (KDIGO) Acute Kidney Injury Work Group (2012). KDIGO clinial practice guideline for acute kidney injury. Kidney Int Suppl.

[9] Rewa O, Bagshaw SM (2014). Acute kidney injury: epidemiology, outcomes ad economics. Nat Rev Nephrol.

[10] Bagshaw SM, George C, Dinu I, Bellomo R (2008). A multi-centre evaluation of the RIFLE criteria for erly acute kdney injury in critically ill patients. Nephrol Dial Transplant.

[11] Ostermann M, Chang R (2007). Acute kidney injury in the intensive cara unit according to RIFLE. Crit Care Med.

[12] Skinner DL, Harcastle TC, Rodseth RN, Muckart DJJ (2014). The incidence and outcomes of acute kidney injury amongst patiens admitted to a level I trauma unit. Injury Int J Care Injured.

[13] Baitello AL, Marcatto G, Yagi RK (2013). Risk factors for injury acute renal failure in patients with severe trauma and its effect on mortality. J Bras Nefrol.

[14] Nongnuch A, Panorchan K, Davenport A (2014). Brain-kidney crosstalk. Crit Care.

[15] Regel G, Lobenhoffer P, Grotz M, Pape HC, Lehmann U, Tscherne H (1995). Treatment results of patients with multiple trauma: an analysis of 3406 cases treated between 1972 and 1991 at German Level I Trauma Center. J Trauma.

[16] Vivino G, Antonelli M, Mro ML, Cottini F, Conti G, Bufi M (1998). Risk factors for acute renal failure in trauma patients. Intensive Care Med.

[17] Swaroop M, Siddiqui SM, Sagar S, Crandall ML (2014). The problem of the pillion rider: India’s helmet law and New Delhi’s exemption. J Surg Res.

[18] Fang L, You H, Chen B, Xu Z, Gao L, Liu J (2010). Mannitol is an independent risk factor of acute kidney injury after cerebral trauma: a case–control study. Ren Fail.

